# Laboratory-developed tests and in vitro diagnostics: A regulatory overview for anatomic pathology

**DOI:** 10.1093/ajcp/aqae181

**Published:** 2025-02-06

**Authors:** Jonathan R Genzen, Lauren J Miller, Anton V Rets, Kajsa E Affolter

**Affiliations:** Department of Pathology, University of Utah Health, Salt Lake City, UT, US; ARUP Laboratories, Salt Lake City, UT, US; Department of Pathology, Michigan Medicine, Ann Arbor, MI, US; Department of Pathology, University of Utah Health, Salt Lake City, UT, US; ARUP Laboratories, Salt Lake City, UT, US; Department of Pathology, University of Utah Health, Salt Lake City, UT, US; ARUP Laboratories, Salt Lake City, UT, US

**Keywords:** anatomic pathology, in vitro diagnostics, laboratory-developed tests, Food and Drug Administration, Clinical Laboratory Improvement Amendments, laboratory regulations

## Abstract

**Objectives:**

The US Food and Drug Administration’s Final Rule on laboratory-developed tests was published on May 6, 2024. The objective of this article is to explain the Final Rule and existing in vitro diagnostic regulations in the context of anatomic pathology.

**Methods:**

The Final Rule, US in vitro diagnostic regulations, guidance documents, government publications, websites, news articles, and publications were reviewed, with sources including the *Federal Register*, the Code of Federal Regulations, the US Code, statutory text, PubMed, and Internet resources. Regulations applicable to device classifications and product codes relevant to anatomic pathology were highlighted.

**Results:**

The Final Rule outlines requirements and enforcement discretion policies applicable to anatomic pathology, including the Food and Drug Administration’s targeted enforcement discretion for “1976-type” laboratory-developed tests and partial enforcement discretion with laboratory-developed tests for unmet needs. Existing regulations, including the classification and requirements applicable to Class I, II, and III medical devices, are reviewed, including those for immunohistochemistry kits and reagents, analyte specific reagents, and research use only reagents and equipment.

**Conclusions:**

Pathologists, laboratory directors, managers, and supervisors responsible for anatomic pathology testing should be familiar with existing regulations and the Final Rule to ensure compliance with federal laws and regulations.

**Key Points**

- The US Food and Drug Administration’s (FDA’s) 2024 Final Rule on laboratory-developed tests (LDTs) expanded its authority to include when an assay manufacturer is a clinical laboratory.
- LDT Final Rule enforcement discretion policies for 1976-type LDTs and testing for unmet needs are particularly relevant for anatomic pathology.
- Immunohistochemistry kits and reagents are categorized by the FDA as Class I, II, or III in vitro diagnostics depending on their use.

## A BRIEF HISTORY OF IN VITRO DIAGNOSTIC AND LABORATORY-DEVELOPED TEST REGULATION

### Food and Drug Administration and Centers for Medicare & Medicaid Services Authority

The US Food and Drug Administration (FDA) and the Centers for Medicare & Medicaid Services (CMS) are separate agencies of the US Department of Health and Human Services within the executive branch of the federal government, and both serve regulatory roles in the oversight of clinical laboratory diagnostic assays and testing. The FDA’s regulatory authority over medical devices used in the “diagnosis, cure, mitigation, [and] treatment” of disease is based on the Federal Food, Drug, and Cosmetic Act (FD&C Act) passed by Congress in 1938.^1^ This legislation was further amended by the Medical Device Amendments of 1976 (the MDA), which established the modern risk-based framework for in vitro diagnostic (IVD) regulation.^2^ More specifically, the MDA clarified the agency’s authority to regulate reagents and instruments used for the diagnosis of disease when distributed through interstate commerce.^1^ Under the MDA, the FDA has oversight authority over the safety and effectiveness of IVDs and the quality of IVD design and manufacture.^3^ This regulatory framework is designed to assess both analytical validity and clinical validity of IVDs before their commercial distribution.

CMS is the federal agency primarily responsible for the implementation of the Clinical Laboratory Improvement Amendments of 1988 (CLIA),^4^ which was enacted as an amendment to the Public Health Services Act of 1944, and it revised and expanded the federal program for the certification and oversight of all human clinical laboratory diagnostic testing conducted in the United States.^4,5^ Through CLIA, CMS provides laboratory certification (see 42 USC § 263a) and oversees the quality of the clinical testing process, the quality of the laboratory, and the requirements to assess laboratory performance.^3^ CLIA regulations also include standards for performance specifications related to analytical validity of both FDA-cleared/approved IVDs and laboratory-developed tests (LDTs) prior to a laboratory reporting patient results (see 42 C.F.R. § 493.1253 [b]).

### LDT Oversight

LDTs predate the modern commercial IVD industry, and as such, clinical laboratories have developed and performed LDTs long before the enactment of either the MDA or CLIA. It is pertinent to note that LDTs were not mentioned in the MDA, nor were they discussed in the associated congressional hearings before its enactment.^6^ The FDA first acknowledged learning about the existence of LDTs in a 1992 draft compliance policy guide.^7^ LDTs are also not specifically mentioned in the CLIA statute, although written testimony from a New York state official provided in a 1988 congressional hearing before CLIA’s enactment noted that laboratories that “use their own techniques and reagents” do not need approval from the FDA.^6,8^

LDTs were directly addressed as federal CLIA regulations were finalized in 1992 (and updated in 2003) regarding performance standards required when a clinical laboratory “modifies an FDA-cleared or approved test system, or introduces a test system not subject to FDA clearance or approval (including methods developed in-house and standardized methods such as text book procedures)” (see 42 C.F.R. § 493.1253 [b][2]).^6^ Clinical laboratories have been performing LDTs under these federal CLIA regulations since that time. While the CLIA statute and subsequent corresponding regulations do not specifically include clinical validity requirements related to LDTs, both a CLIA-deemed accreditation organization (ie, the College of American Pathologists Laboratory Accreditation Program^9^) and a CLIA-exempt state licensure program (ie, New York State Department of Health Clinical Laboratory Evaluation Program [NYS CLEP]^10^) do include clinical validity requirements as part of their CLIA accreditation program/clinical laboratory standards.

In 2010—more than three decades after the enactment of the MDA—the FDA formally announced that it intended to regulate LDTs as IVDs. The FDA asserted that it would discontinue a purported policy of LDT enforcement discretion. In general terms, enforcement discretion can be described as a federal agency asserting that it has the right to regulate something but chooses to not do so. In its proposed plan, the FDA said it would end its asserted enforcement discretion over LDTs and alternatively begin exercising regulatory oversight over them in the future. A corresponding LDT regulatory framework was developed over the next several years under a draft guidance rulemaking approach and was shared by the FDA in 2014.^11^ This 2014 draft oversight framework generated significant attention (both positive and negative) from the clinical laboratory, IVD, and patient care communities.

In 2015, the FDA released a controversial report immediately before a House of Representatives Energy and Commerce Committee, Subcommittee on Health hearing that served to support the FDA’s justification for increased LDT oversight.^12^ Within this report, the FDA provided 20 examples of LDTs (either specific test kits or multiple tests for the same diagnostic category), which it claimed to be associated with actual or potential harm to patients (eg, by providing false-positive or false-negative results). In this report, the FDA advocated for an oversight framework “that is appropriately tailored so that it is complementary and does not duplicate the oversight currently provided under CLIA.”^12^ An external analysis critical of this report was subsequently released by the Association for Molecular Pathology.^13^ The FDA ultimately discontinued its draft guidance plans and withdrew the proposed LDT oversight framework immediately after the 2016 presidential election, and the agency highlighted its existing thinking on LDTs in a discussion paper released shortly before the presidential inauguration in January 2017.

LDT and IVD regulatory reform efforts then shifted toward Congress, where two competing potential bills related to laboratory diagnostics—the Verifying Accurate Leading-edge IVCT Development Act (VALID Act)^14^ and the Verified Innovative Testing in American Laboratories Act (VITAL Act)^15^—were subsequently introduced. The VALID Act would have created a new regulatory oversight structure under the FDA covering both IVDs and LDTs as in vitro clinical tests, and this would have also subjected clinical laboratories performing LDTs to FDA registration and user fees. Alternatively, the VITAL Act would have clarified that regulation of LDTs should be under the CMS and not the FDA. While neither bill was enacted by the 117th Congress (2021-2022 session), the VALID Act did receive significant attention during the 2022 Medical Device User Fee Amendments (MDUFA) negotiations and in deliberations leading up the 2023 Consolidated Appropriations Act.

## THE LDT FINAL RULE

### Development and Release

After Congress declined to advance either the VALID Act or the VITAL Act, the FDA signaled in early 2023 that it intended to move forward with regulating LDTs through a notice and comment rulemaking approach. Under requirements established by the Administrative Procedure Act, through notice and comment rulemaking, a federal agency must notify the public of a new proposed regulation, allow the public the opportunity to provide feedback and comments to the agency, and then consider this feedback before advancing the final regulation.^16^ As such, the FDA published its proposed rule for LDT oversight on October 3, 2023^17,18^; provided a 60-day public comment period (notably declining requests for an extension, including a cosigned request from 89 patient advocacy organizations, professional societies, and health care organizations); and released their final rule on May 6, 2024.^19^ We will refer to this as the “LDT Final Rule” throughout the remainder of this article. The FDA’s accelerated schedule for such consequential rulemaking was widely regarded as an attempt by the agency to minimize the possibility of congressional review of the LDT Final Rule under timelines and procedures established by the Congressional Review Act. The LDT Final Rule includes an extensive preamble and hundreds of responses to general categories of public comments that provide insight into the FDA’s perspective on a variety of topics. For readers who are more interested in a shorter guide to the LDT Final Rule, the FDA released a 21-page small entity compliance guide in June 2024.^20^ Consideration of litigation against the LDT Final Rule and the impact of the recent 2024 US presidential and congressional elections is presented in the Discussion section at the end of this article.

### Summary of Contents

The LDT Final Rule is essentially a 10-word addition to the definition of “in vitro diagnostic products,” a legal definition that the FDA uses to encompass “reagents, instruments, and systems intended for use in the diagnosis of disease” (see 42 C.F.R. § 809.3 (a)). Through the LDT Final Rule, this definition was expanded to clarify that this also means “including when the manufacturer of these products is a laboratory.”^21^ Through this 10-word addition, the FDA asserts that the existing regulatory framework for commercially manufactured IVDs (ie, physical products) now also applies to all LDTs in clinical laboratories.

In the LDT Final Rule preamble, the FDA also asserts that it is ending its policy of “general enforcement discretion” over LDTs (ie, it will begin regulating them), but it will also adopt new “targeted enforcement discretion” for certain types and settings of clinical laboratory testing. For example, the FDA states that it does not intend to regulate LDTs used in Veterans Health Administration and Department of Defense settings.^22^ It also does not intend to regulate LDTs used for forensic (ie, law enforcement) purposes or human leukocyte antigen testing for transplant.^22^ The FDA also does not intend to regulate “1976-type LDTs,” which it describes as LDTs using manual techniques (without automation or software) and with components legally marketed for clinical use (eg, no research use only [RUO] reagents) when they are “designed, manufactured, and used” within a single CLIA high-complexity laboratory setting.^23^ For the types and settings of LDTs described in this paragraph, none of the additional LDT Final Rule requirements will apply. However, it is important to emphasize that the policies of targeted and partial enforcement discretion are not actually codified in the LDT Final Rule. Thus, the FDA can, at its discretion, decide to discontinue and/or modify enforcement discretion at any time. Furthermore, the testing that falls under this targeted enforcement discretion represents a small fraction of the activities conducted by most anatomic pathology laboratories.

The FDA also announced in the preamble to the LDT Final Rule that certain categories of “partial enforcement discretion”—where some (but not all) of the newly announced LDT regulations apply—will also exist. Categories of partial enforcement discretion include LDTs that were currently marketed as of the May 6, 2024, LDT Final Rule publication date, testing for rare red blood cell antigens, testing for unmet needs in some circumstances (including when the LDT is “manufactured and performed by a laboratory integrated within a healthcare system to meet an unmet need of patients receiving care within the same healthcare facility”),^24^ and for LDTs that have been approved by NYS CLEP.^22^ The FDA outlines which general requirements apply to which categories of partial enforcement discretion in a table located on its LDT website.

### Timeline and “Phase-Out” Policy

**FIGURE 1:**
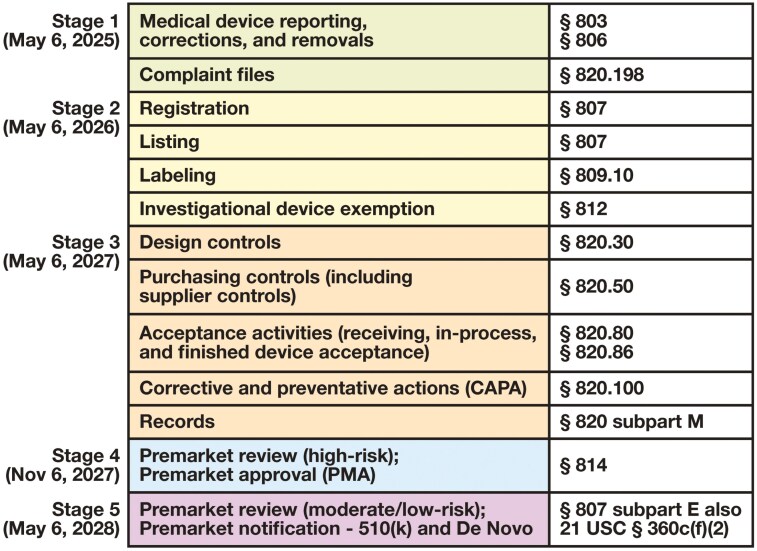
LDT Final Rule timeline of requirements. Requirements by stage are outlined by due date. Associated requirements are referenced to their location in the US Code of Federal Regulations. For example, medical device reporting (§ 803) is a reference to 21 C.F.R. § 803, also located at https://www.ecfr.gov/current/title-21/chapter-I/subchapter-H/part-803. LDT, laboratory-developed test.

The LDT Final Rule requirements are delineated in a 5-stage “phase-out” policy (ie, “phasing out” enforcement discretion) occurring over a 4-year period, as outlined in **FIGURE 1**.^25,26^ Briefly, stage 1 requirements (due May 6, 2025) include medical device reporting (MDR), device correction and removal requirements, and complaint file handling. Both MDR and compliant file regulations specifically include requirements for written procedures. While often discussed in the context of corrections and removals, the FDA’s authority to recall problematic IVDs is not codified within 21 C.F.R. § 806. Readers interested in learning more about recall processes and authorities should see 21 C.F.R. § 7 and 21 C.F.R. § 810.

Compliance with stage 2 is due May 6, 2026, and includes registration with the FDA, listing of LDTs with the FDA, submission of LDT labeling (as applicable), and compliance with investigational device exemption requirements. Compliance with stage 3 is due on May 6, 2027, and includes adherence with a subset of FDA quality system (QS) regulations (ie, current good manufacturing practice [CGMP]), including design controls, purchasing and supplier controls, acceptance activities, corrective and preventative actions, and record requirements. Compliance with stage 4 is due on November 6, 2027, and includes submission of new high-risk LDTs for premarket review through the premarket approval (PMA) process, whereas compliance with stage 5 is due on May 6, 2028, and includes submission of new moderate-risk (and potentially low-risk) LDTs for premarket review through the premarket notification 510(k), or De Novo pathways, as applicable. New LDTs that have been developed or existing LDTs that have undergone significant modifications since the publication of the LDT Final Rule (May 6, 2024) will be subject to these premarket review requirements.

### 1976-Type LDTs

**FIGURE 2:**
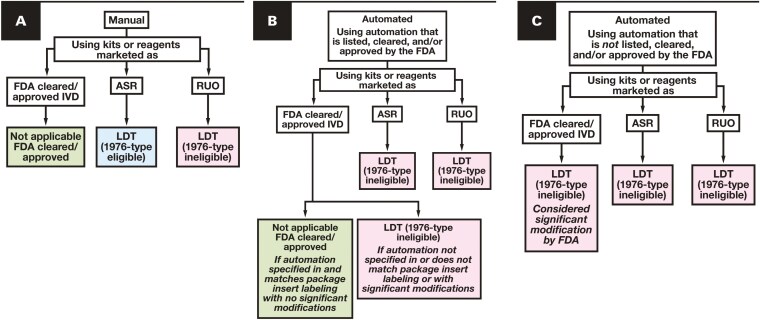
1976-type LDT eligibility for immunohistochemistry and in situ hybridization. 1976-type eligibility status under the LDT Final Rule according to (**A**) manual testing status, (**B**) automated testing status (using automation that is listed, cleared, and/or approved by the FDA), and (**C**) automated testing status (using automation that is *not* listed, cleared, and/or approved by the FDA), based on whether the assay uses FDA-cleared/approved, ASR, or RUO reagents. ASR, analyte-specific reagent; FDA, US Food and Drug Administration; IVD, in vitro diagnostic; LDT, laboratory-developed test; RUO, research use only.

The 1976-type LDT category of targeted enforcement discretion in the LDT Final Rule has generated significant attention across the clinical laboratory community, as it is essentially an exemption category for LDT Final Rule requirements. Relevant to anatomic pathology, the FDA has included manual immunohistochemistry (IHC) using legally marketed reagents (ie, not RUO reagents) as well as other manual testing, including “tests that use staining antibodies,” “general purpose reagents for cytology, hematology,” “karyotypic” tests, and “fluorescence in situ hybridization” as 1976-type LDTs.^23^ The FDA specifically responded to a public commentor who asked about the exclusion of automation from 1976-type LDTs in the LDT Final Rule, with the commentor noting the paradox of the FDA’s perspective on manual testing, given that “the technical aspect of immunohistochemistry is virtually always automated these days, while interpretation is manual.”^23^ However, the FDA reiterated their original position in their response, noting that the FDA does not believe that expanding the 1976-type definition to include the use of automation is appropriate.^23^ This highlights the arbitrary application of requirements across the LDT Final Rule. While scientific discoveries involving the use of antibodies in tissue staining certainly occurred before 1976, subsequent practical advances and widespread adoption of IHC in the clinical laboratory community occurred well after 1976. For instance, heat-induced epitope retrieval, described in the 1990s, allowed identification of a myriad of antigens in formalin-fixed, paraffin-embedded tissues and revolutionized the clinical utility of IHC.^27^ Introduction of extra steps to IHC protocols added complexity and made manual IHC very challenging in a clinical setting. The advantages of automated staining (eg, higher reproducibility, greater testing volumes) made automation virtually ubiquitous.^28^ Regardless, testing that incorporates automation, software, or RUO reagents cannot qualify under the FDA’s 1976-type targeted enforcement discretion category, and consequently, most clinically performed IHC and in situ hybridization tests will be excluded from using this category. An illustration summarizing the authors’ understanding of 1976-type LDT eligibility of IHC kits and reagents and in situ hybridization assays is provided in **FIGURE 2**.

### Unmet Needs

While the unmet needs provision in the LDT Final Rule was likely created as an attempt to address widespread concerns about test availability for rare disorders, the FDA has added significant limitations on the use of the unmet needs provision in many circumstances. For example, the unmet needs provision cannot be used for patients “treated at an affiliated hospital with different corporate ownership”^29^ (ie, outreach testing), it is “limited to LDTs that are ordered by a healthcare practitioner on the staff or with credentials and privileges at a facility owned and operated by the same healthcare system employing the laboratory director and performing the LDT,”^29^ and the FDA specifically notes that performance improvements or lower cost do not qualify as unmet needs.^30^ The unmet needs provision is therefore also not applicable in most reference laboratory settings that are commonly used in testing for rare disorders. In the context of anatomic pathology, the unmet needs provision may be useful to laboratories that are offering LDTs (and/or using analyte specific reagent [ASR] or RUO reagents) for testing within their health system when no commercially available FDA-cleared/approved reagents are available to meet the clinical need that such an assay would otherwise provide or when the introduction of automation in this circumstance (or RUO reagents) would prevent application of a 1976-type targeted exemption. As the FDA intends to exercise partial enforcement discretion in this category, the laboratory should be aware that under the LDT Final Rule, stage 1, stage 2, and the records requirement in stage 3 still apply under the unmet needs provision. Additionally, as soon as FDA-cleared/approved reagents (eg, IHC or flow cytometry antibodies) become available, the unmet needs provision can no longer be used by a clinical laboratory.

### Test Modifications

The FDA provided general information on test modifications in the LDT Final Rule that should be considered by all clinical laboratories. The FDA expects compliance with QS (stage 3) and premarket review (stages 4/5) requirements for currently marketed LDTs when certain types of modifications are made. The FDA specifies that such “significant” modifications include changes in the indications for use, changes in the operating principle, use of significantly different technologies (including a change from manual to automated methods or the introduction of artificial intelligence/machine learning), and any changes that adversely affect an LDT’s safety or performance.^31^ The FDA does not intend to require premarket review for more minor modifications to another manufacturer’s 510(k) or De Novo authorized test, although they will require premarket review for modifications to tests that have Class III premarket approvals (ie, high-risk tests).^32^

In anatomic pathology, it is likely that the FDA has significantly underestimated the amount of assay optimization/modification required to obtain adequate performance using FDA-cleared/approved reagents. Standard good laboratory practice involves periodically verifying the performance of IHC and making necessary adjustments, such as with new reagent lots, which may show between-lot variation, requiring possible changes in antibody dilution, incubation, retrieval times, addition of blockers and enhancements, and so on.^33^ Variability of specimen processing (fixation, decalcification) also may necessitate adjustments of the recommended protocol. For context, in its Guidance for Submission of Immunohistochemistry Applications to the FDA published in 1998, the FDA describes content requirements for manufacturers related to package insert procedures, including instructions for optimization, fixation, processing, antigen recovery, check board titration, and storage and handling of undiluted and diluted reagents.^34^ However, the agency in that guidance states that “such instructions will enable the end user to follow manufacturer’s instructions for almost all circumstances that arise with the use of this IHC . . . if the manufacturer’s recommendations are followed without major modification.”^34^ One interpretation of the LDT Final Rule could be that such modifications may not be considered “significant” or “major” by the FDA (eg, would not require premarket review), although stage 1 requirements, stage 2 requirements, and the records requirement of stage 3 for such “minor” modifications going forward would still apply. The clinical laboratory community would benefit tremendously if the FDA clarified what types of modifications are considered minor. It should also be noted that the FDA stated in a September 2024 webinar that it does not anticipate requiring submission of labeling for certain modifications to other manufacturers’ 510(k) and De Novo authorized tests, although such clinical laboratories would still be required to remain compliant with labeling and to maintain such documents within the laboratory’s files.^35^ Clarification on whether and/or which minor (routine) test modifications are subject to FDA correction and removal reporting requirements (ie, because they improve assay performance and by definition reduce risk to public health) would also be helpful to the clinical laboratory community as it prepares for the LDT Final Rule.

## FDA REGULATION OF IN VITRO DIAGNOSTICS

Developing an optimal strategy for how to effectively comply with the LDT Final Rule going forward—in all clinical laboratories performing LDTs, including anatomic pathology—requires an understanding of the existing regulatory framework for IVDs. The following section describes how the FDA classifies medical devices, as well as what requirements specifically apply to different classes and product categories of IVDs. Examples of devices used in anatomic pathology are highlighted to illustrate the types of requirements that will apply to new assays that do not fall under targeted or partial enforcement discretion.

### Medical Device Classification

The risk-based structure of medical device regulation was introduced as Section 513 of the FD&C Act by the MDA in 1976, and it has been incorporated in the US code of law (see 21 USC § 360c). This law and the accompanying federal regulations outline three classes of devices that are intended to correlate with risk. All Class I, II, and III devices are subject to FDA QS/CGMP regulations **TABLE 1**, unless they are specifically exempt from certain requirements.

**TABLE 1:** FDA Quality System (QS) Regulation/Medical Device Current Good Manufacturing Practices (CGMP) Requirements^a^

Subpart A—General Provisions
§ 820.1 Scope
§ 820.3 Definitions
§ 820.5 Quality system
Subpart B—Quality System Requirements
§ 820.20 Management responsibility^b^
§ 820.22 Quality audit^b^
§ 820.25 Personnel
Subpart C—Design Controls
§ 820.30 Design controls^c^
Subpart D—Document Controls
§ 820.40 Document controls^b^
Subpart E—Purchasing Controls
§ 820.50 Purchasing controls^c^
Subpart F—Identification and Traceability
§ 820.60 Identification
§ 820.65 Traceability
Subpart G—Production and Process Controls
§ 820.70 Production and process controls
§ 820.72 Inspection, measuring, and test equipment
§ 820.75 Process validation
Subpart H—Acceptance Activities
§ 820.80 Receiving, in-process, and finished device acceptance^c^
§ 820.86 Acceptance status^c^
Subpart I—Nonconforming Product
§ 820.90 Nonconforming product
Subpart J—Corrective and Preventive Action
§ 820.100 Corrective and preventive action^c^
Subpart K—Labeling and Packaging Control
§ 820.120 Device labelling
§ 820.130 Device packaging
Subpart L—Handling, Storage, Distribution, and Installation
§ 820.140 Handling
§ 820.150 Storage
§ 820.160 Distribution
§ 820.170 Installation
Subpart M—Records^b^
§ 820.180 General requirements^c^
§ 820.181 Device master record^c^
§ 820.184 Device history record^c^
§ 820.186 Quality system record^c^
§ 820.198 Complaint files^c,d^
Subpart N—Servicing
§ 820.200 Servicing
Subpart O—Statistical Techniques
§ 820.250 Statistical techniques

^a^Source: 21 C.F.R. § 820. See https://www.ecfr.gov/current/title-21/chapter-I/subchapter-H/part-820. See also https://www.fda.gov/medical-devices/postmarket-requirements-devices/quality-system-qs-regulationmedical-device-current-good-manufacturing-practices-cgmp.

^b^Even though they are cross-referenced in Subpart M, the US Food and Drug Administration (FDA) does not expect compliance by CLIA laboratories with 21 C.F.R. § 820.20, § 820.22, and § 820.40, as noted in *Fed Reg*. 2024;89(88):37309, footnote 47.

^c^The FDA expects compliance by CLIA laboratories per the LDT Final Rule in stage 3; see *Fed Reg*. 2024;89(88):37309.

^d^Compliance due in stage 1 of the LDT Final Rule.

Risks associated with Class I devices (eg, low risk) are addressed by the FDA through application of “general controls.” General controls include manufacturer requirements related to device adulteration, misbranding, registration and listing, banned devices, notifications, records and reports, and other general provisions (see **TABLE 2**).^36^ The FDA’s 1998 final rule on IHC device classification/reclassification emphasized the FDA’s intention to classify most IHC reagents and kits as Class I, including those “used to classify tumors.”^37^ Class I devices are subject to FDA registration and device listing requirements, for example, but not FDA premarket notification 510(k) or PMA requirements. Several types of common reagents and equipment used in anatomic pathology are considered Class I, including general purpose reagents, dye and chemical solution stains, tissue-processing equipment, devices for sealing microsections, microscopes and accessories, automated slide stainers, and automated tissue processors (see **TABLE 3**, **TABLE 4**, and TABLE 5). There are also Class I designations for certain ASRs and IHC kits and devices, as described below.

**TABLE 2:** General Controls^a^

Category	FD&C Act section	US Code
Adulterated Drugs and Devices	501	21 USC § 351
Misbranded Drugs and Devices	502	21 USC § 352
Registration of Producers of Drugs or Devices	510	21 USC § 360
Banned Devices	516	21 USC § 360f
Notifications and Other Remedies	518	21 USC § 360h
Records and Reports on Devices	519	21 USC § 360i
General Provisions Respecting Control of Devices Intended for Human Use	520	21 USC § 360j

FD&C Act, Federal Food, Drug, and Cosmetic Act.

^a^Source: FD&C Act Chapter V: Drugs and Devices. https://www.fda.gov/regulatory-information/federal-food-drug-and-cosmetic-act-fdc-act/fdc-act-chapter-v-drugs-and-devices. Accessed December 20, 2024. See also https://www.fda.gov/medical-devices/overview-device-regulation/regulatory-controls. Accessed January 3, 2025.

**TABLE 3:** Examples of General Purpose Reagents^a^

Cytological preservatives
Decalcifying reagents
Fixatives and adhesives
Tissue-processing reagents
Isotonic solutions and pH buffers
Thermus aquaticus polymerase
Substrates for enzyme immunoassay

^a^Source: 21 C.F.R. § 864.4010.

**TABLE 4:** Product Codes Listed Under the Immunohistochemistry Reagents and Kits Device Regulatory Classification (21 C.F.R. § 864.1860)^a^

Product code	Device name	Device class	Third party review eligible
MXZ	Immunohistochemistry Assay, Antibody, Progesterone Receptor	II	Y
MYA	Immunohistochemistry Antibody Assay, Estrogen Receptor	II	Y
NBK	System, Test, (IHC), Tumor Marker, Monitoring, Bladder Cancer	II	N
NJT	Immunohistochemistry Reagents and Kits	I	N
NJW	Control Material, Her-2/Neu, Immunohistochemistry	II	Y
NKF	Immunohistochemistry Antibody Assay, C-Kit	III	N
NOT	Microscope, Automated, Image Analysis, Operator Intervention	II	N
NQF	Immunohistochemistry Assay, Antibody, Epidermal Growth Factor Receptor	III	N
NQN	Microscope, Automated, Image Analysis, Immunohistochemistry, Operator Intervention, Nuclear Intensity & Percent Positivity	II	N
NTR	Immunohistochemical Reagent, Antibody (Monoclonal or Polyclonal) to P63 Protein in Nucleus of Prostatic Basal Cells	I	N
OEO	Automated Digital Image Manual Interpretation Microscope	II	N
QNH	Immunohistochemistry Test, DNA Mismatch Repair (MMR) Protein Assay	III	N
QZJ	Immunohistochemistry Assay, Antibody, Claudin 18	III	N
QUL	Immunohistochemistry Assay, Antibody, Folr1	III	N

IHC, immunohistochemistry; N, no; Y, yes.

^a^Please see US Food and Drug Administration (FDA) website (https://www.accessdata.fda.gov/scripts/cdrh/cfdocs/cfpcd/classification.cfm) for updated product codes and classifications. The download files option on FDA website provides a complete list of product codes that is updated weekly and can be imported into Excel. Product codes LPI and LPJ were omitted from the table due to chemistry review panel classification status.

The risks associated with Class II devices (eg, moderate risk) are addressed by the FDA through application of both “general controls” and “special controls.” Special controls may include a variety of additional requirements for specific devices such as the use of performance standards, postmarket surveillance, patient registries, special labeling requirements, premarket data requirements, and/or adherence to various guidelines.^36^ Class II devices are subject to the premarket notification 510(k) process to receive FDA clearance before commercial distribution. Through the 510(k) process, a new device must be determined to be “substantially equivalent” to a device already cleared by the FDA.

For higher-risk (Class III) devices, the FDA does not consider general and special controls alone as sufficient to ensure the safety and effectiveness of a device. Therefore, the FDA requires a PMA process with review of extensive submitted information to ensure device safety and effectiveness before commercial distribution. This review typically includes submission of clinical validation data. Devices that do not already have a predicate device to compare to are also, by default, considered Class III devices. There is now a separate and less expensive De Novo classification request process, however, for a manufacturer to request that a novel device should not be automatically classified as a Class III device but rather should be subject to either Class I or Class II requirements.^40^ The FDA has recently announced its intention to reclassify most existing Class III IVDs as Class II IVDs before stage 4 of the LDT Final Rule goes into effect.^32,41^ This multiyear initiative would decrease some of the administrative cost and burden to clinical laboratories, but lack of definitive clarity on device classification until immediately before an LDT Final Rule deadline makes planning activities particularly challenging.

### General Purpose Reagents

A general purpose reagent is defined as “a chemical reagent that has general laboratory application, that is used to collect, prepare, and examine specimens from the human body for diagnostic purposes, and that is not labeled or otherwise intended for a specific diagnostic application,” and general purpose reagents may also include “labware or disposable constituents of tests” but not “laboratory machinery, automated or powered systems” (see 21 C.F.R. § 864.4010). Examples of general purpose reagents listed in the Code of Federal Regulations are included in **TABLE 3**. General purpose reagents are Class I devices, and as long as they are not labeled as sterile, they are exempt from FDA QS/CGMP regulations, with the exception of records and complaint file requirements (see 21 C.F.R. § 864.4010).

### Dye and Chemical Solution Stains

Dye(s) and chemical solution stains are defined in the Code of Federal Regulations as “mixtures of synthetic or natural dyes or nondye chemicals in solutions used in staining cells and tissues for diagnostic histopathology, cytopathology, or hematology” (see 21 C.F.R. § 864.1850). This definition encompasses many of the common materials found in a conventional histopathology laboratory, such as routine hematoxylin and eosin stains, as well as special stains like trichrome, iron, and Grocott-Gömöri’s methenamine silver, among others, and are considered Class I devices subject to general controls.

### ASRs

Analyte specific reagents are defined as “antibodies, both polyclonal and monoclonal, specific receptor proteins, ligands, nucleic acid sequences, and similar reagents which, through specific binding or chemical reaction with substances in a specimen, are intended for use in a diagnostic application for identification and quantification of an individual chemical substance or ligand in biological specimens.”^39^ The FDA has more generally described ASRs as the “building blocks” or “active ingredients” of IVDs and/or LDTs, and a frequently asked questions guidance document on ASRs was published by the FDA in 2007 and is summarized below.^39^ The FDA has provided the following general characteristic of ASRs, stating that they are (1) used to detect a single ligand or target, (2) not labeled with instructions for use or performance claims, and (3) not promoted for use on specific designated instruments or in specific tests.^39^ Many IHC test kit inserts do not specify the platform for performing the IHC, thereby potentially classifying the process as an LDT when automation is introduced.

An FDA final rule on ASR classification was published in 1997.^42,43^ Most ASRs are considered Class I devices, but select types of ASRs also are Class II—for example, those “used in blood banking tests . . . (eg, certain cytomegalovirus serological and treponema pallidum nontreponemal test reagents)”—whereas others are Class III (eg, those for HIV and tuberculosis diagnosis or those used for donor screening; see 21 C.F.R. § 864.4020 for additional details).^39,42^ Analyte specific reagents are subject to general controls (including the QS/CGMP requirements described in 21 C.F.R. Part 820; see **TABLE 1**). Along with requirements outlined in 21 C.F.R. § 864.4020, ASRs have certain restrictions on their sale, distribution, and use to IVD manufacturers and high-complexity CLIA laboratories when used for clinical diagnostic purposes (21 C.F.R. § 809.30), as well as labeling requirements specific to ASRs (21 C.F.R. § 809.10(e)). Advertising and promotional materials for ASRs may not include any “claims for clinical or analytical performance.”^39^

### RUO and Investigational Use Only (IUO) Reagents

The FDA investigational device exemption requirements are listed at 21 C.F.R. Part 812. The FDA describes an RUO product as “an IVD product that is in the laboratory research phase of development and is being shipped or delivered for an investigation that is not subject to part 812,” whereas investigational use only (IUO) reagents are products that are in “the product testing phase of development.”^38^ There are specific labeling requirements for RUO products (eg, “For Research Use Only”)^38^; otherwise, the FDA would consider the RUO to be misbranded. Since RUO and IUO products are intended for research and/or investigation (and not in vitro diagnostic use), they are not subject to FDA QS/CGMP requirements.

In the LDT Final Rule, the FDA acknowledges that RUO reagents may continue to be used as components of LDTs (except in 1976-type LDTs under targeted enforcement discretion).^44^ However, as these reagents were not produced by manufacturers under the FDA QS/CGMP framework, laboratories using RUOs in LDTs are subject to FDA QS purchasing control requirements (21 C.F.R. § 820.50) to ensure that laboratories “assess the capability of their suppliers to produce acceptable components”^45^ and that “the laboratory is responsible for qualifying such components in its IVDs.”^44^

### IHC Kits and Reagents

IHC kits and reagents are among the most important types of assays used in anatomic pathology. The FDA has defined an IHC device as “an in vitro diagnostic reagent or test kit that uses immunological methods to identify antigens in tissues or intact cells,” whereas an IHC reagent is “the primary antibody of an IHC assay that is developed to specifically target, react to, or combine with, a particular cellular or tissue constituent, or antigen, using specific immunological characteristics of the antibody.”^37^ In 1998, the FDA released a final rule on the classification/reclassification of IHC reagents and kits and how they should be assigned to the three medical device classes described above.^37^ As noted in its proposed rule two years earlier, the FDA initiated this classification process so that IHC reagents and kits introduced before and after 1976 would be regulated using a more consistent approach.^46^ For example, between 1976 and the 1998 final rule, all new IHC reagents and kits were being automatically classified as high-risk Class III devices, regardless of potential risk to patients. The 1998 final rule classified and/or reclassified existing and future IHC devices into the framework described below.

Class I IHC reagents and kits were defined as those that “provide the pathologist with adjunctive diagnostic information that may be incorporated into the pathologist’s report, but that is not ordinarily reported to the clinician as an independent finding,” noting that “these IHC’s are used after the primary diagnosis of tumor (neoplasm) has been made by conventional histopathology using nonimmunologic histochemical stains, such as hematoxylin and eosin” (see 21 C.F.R. § 864.1860). The 1998 final rule further describes these as “adjunctive tests [to conventional histopathologic diagnostic examination] and presenting a low risk to public health.”^37^ An example of a Class I IHC specifically mentioned in the IHC reagents and kit regulation is keratin (see 21 C.F.R. § 864.1860). Many other Class I IHC reagents and kits can be found by searching for the “NJT” product code on the FDA establishment registration and device listing database.^47^ These IVDs are exempt from premarket notification/510(k) submission, although they are still subject to FDA general controls and QS/CGMP requirements. Most IHC stains used by anatomic pathologists are considered diagnostic markers and therefore would be categorized as Class I by the FDA.

Class II IHC reagents and kits are defined as those that “are intended for the detection and/or measurement of certain target analytes in order to provide prognostic or predictive data that are not directly confirmed by routine histopathologic internal and external control specimens” and that “provide the pathologist with information that is ordinarily reported as independent diagnostic information to the ordering clinician, and the claims associated with these data are widely accepted and supported by valid scientific evidence” (see 21 C.F.R. § 864.1860). The most widely recognized Class II IHC kits and reagents are for estrogen receptor and progesterone receptor. Many IHC labs, serving both small and large hospital systems, offer the abovementioned markers. Additionally, many of these tests have an FDA clearance designated for a specific automated platform. As with other Class II IVDs, Class II IHC reagents and kits are subject to both general controls and special controls,^36^ as well as QS/CGMP requirements. The special control for Class II IHC is adherence to the 1998 FDA Guidance for Submission of Immunohistochemistry Applications to the FDA.^34^ This guidance, for example, outlines the “types and amounts” of validation and scientific evidence to support IHC performance claims and templates that should be used by IHC kit and reagent manufacturers for package insert labeling.^34^

Class III IHC reagents and kits are defined as IHCs “intended for any use not described” under the Class I or Class II provisions. Consistent with the PMA requirements for all Class III IVDs,^36,48^ “manufacturers of these IHC’s must submit valid scientific evidence to support the new intended uses.”^37^ While not technically the subject of the 1998 IHC special controls guidance document mentioned above, this guidance does share the FDA’s perspective on Class III IHCs as those that “do not meet the criteria for class 1 or 2, or are IHC’s that meet those criteria but raise new issues of safety and effectiveness” with an example of “markers used to identify new target analytes in tissues that are claimed to be clinically significant genetic mutations and that cannot be confirmed by conventional histopathologic internal and external controls specimens.”^34^ Of note, most companion and complementary diagnostic IHCs are currently classified as Class III devices by the FDA.^49-51^ However, as previously noted, the FDA recently announced that it anticipates most of the existing Class III IVDs will be reclassified as Class II IVDs before stage 4 of the LDT Final Rule goes into effect, but this is not ensured until the reclassification is finalized.^41^ Examples of Class III IHCs include antibody assays for anaplastic lymphoma kinase (ALK), programmed death ligand 1, human epidermal growth factor receptor 2 (HER2/neu), epidermal growth factor receptor, c-kit, Ki-67, DNA mismatch repair assays, claudin 1, and Folr1 (see **TABLE 4**, **Supplementary Appendix S1** [all supplementary material is available at *American Journal of Clinical Pathology* online], the FDA premarket approval database, and the FDA companion diagnostic website^49^). It is noteworthy that antibodies targeting the same antigen may be assigned different classes depending on their intended use. For example, the anti-CD117 (c-kit) antibody may be classified as Class I (if it is used to diagnose a neoplasm, eg, gastrointestinal stromal tumor (GIST)) or Class III (if its expression by GIST must be demonstrated to qualify the patient for imatinib therapy).

The 1998 IHC classification final rule also provides additional perspective on what should be done by manufacturers selling IHC reagents and kits. For example, the FDA notes that if an “IHC primary antibody reagent is sold separately, there should be recommendations for what ancillary reagents and equipment should be used with the IHC reagent to achieve the performance characteristics claimed for the primary IHC reagent.”^52^ However, if “the IHC is marketed as a test kit, there should be performance data with the finished test kit.”^52^ The FDA also states that IHC secondary antibodies are “ancillary” reagents and “are subject to 21 C.F.R. § 864.1860 [ie, immunohistochemistry reagents and kits requirements] only when packaged as components of a complete test system with one or more primary antibodies.”^34^

### In Situ Hybridization Kits and Reagents

There are several product classifications covering in situ hybridization (ISH) kits and reagents pertinent to anatomic pathology (see **Supplementary Appendix S1**). For example, the device categories “fluorescence in situ hybridization (FISH)–based detection of chromosomal abnormalities from patients with hematologic malignancies” and the “system, automated scanning microscope and image analysis for FISH assays” are both classified as Class II. Other Class II ISH devices include FISH probe kits for chronic lymphocytic leukemia and early growth response 1. Some ISH devices that have Class III designations include Her2/Neu, platelet-derived growth factor receptor β polypeptide, TP53 deletion, and ALK.

### Other Common Reagents and Equipment Used in Anatomic Pathology

Along with IHC and ISH reagents and kits, several other broad classifications of reagents and equipment are relevant to anatomic pathology. A general overview of these classifications and applicable requirements is shown in **TABLE 5**. An example of assay product codes under an anatomic pathology-applicable IVD classification is shown in **TABLE 4**. A more comprehensive list of classifications (ie, classification product codes) and subsequent individual product codes of many assays within these broader classifications and applicable to anatomic pathology can be found in **Supplementary Appendix S1**. Device classifications, subsequent product codes, and specific regulatory requirements for each product code can change over time, and readers should therefore consult with the FDA product classification database^53^ and applicable classification regulations^54^ to determine appropriate device classification and product codes before device listing and premarket submissions.

**TABLE 5:** Requirements Across Device Classifications Relevant to Anatomic Pathology^a^

Category	Regulation (21 C.F.R.)	Class	General controls	Special controls	Premarket notification	Premarket approval	QS/CGMP
General Purpose Reagent	§ 864.4010	I	Applies	N/A	Exempt^i^	N/A	Exempt^b^
RUO Reagent	See note^c^	N/A	N/A	N/A	N/A	N/A	N/A
IUO Reagent	See note^c^	N/A	N/A	N/A	N/A	N/A	N/A
ASR	§ 864.4020	I	Applies^d^	N/A	Exempt	N/A	Applies
	§ 864.4020	II	Applies^d^	Applies^e^	Applies	N/A	Applies
	§ 864.4020	III	Applies^d^	N/A	N/A	Applies^f^	Applies
IHC Kit and Reagent	§ 864.1860	I	Applies	N/A	Exempt	N/A	Applies
	§ 864.1860	II	Applies	Applies^g^	Applies^h^	N/A	Applies
	§ 864.1860	III	Applies	N/A	N/A	Applies	Applies
Dye and Chemical Solution Stains	§ 864.1850	I	Applies	N/A	Exempt^i^	N/A	Exempt^j^
Tissue Processing Equipment	§ 864.3010	I	Applies	N/A	Exempt^i^	N/A	Exempt^j^
Device for Sealing Microsections	§ 864.3400	I	Applies	N/A	Exempt^i^	N/A	Applies
Microscopes and Accessories	§ 864.3600	I	Applies	N/A	Exempt^i^	N/A	Exempt^b^
Automated Slide Stainer	§ 864.3800	I	Applies	N/A	Exempt^i^	N/A	Applies
Automated Tissue Processor	§ 864.3875	I	Applies	N/A	Exempt^i^	N/A	Applies
Automated Cell Counter	§ 864.5200	II	Applies	See note^k^	Applies	N/A	Applies
Automated Differential Cell Counter	§ 864.5220	II	Applies	Applies^l^	Applies	N/A	Applies
Flow Cytometric Test System for Hematopoietic Neoplasms	§ 864.7010	II	Applies	Applies^m^	Applies	N/A	Applies
Whole Slide Imaging System	§ 864.3700	II	Applies	Applies^n^	Applies	N/A	Applies
Software Algorithm to Assist Users in Digital Pathology	§ 864.3750	II	Applies	Applies^o^	Applies	N/A	Applies

ASR, analyte specific reagent; C.F.R., Code of Federal Regulations; CGMP, current good manufacturing practices; FDA, US Food and Drug Administration; IHC, immunohistochemistry; IUO, investigational use only; N/A, not applicable; QSR, quality system regulation; RUO, research use only.

^a^This table represents a good-faith attempt at displaying FDA requirements according to medical device classification. Please consult with specific device classification requirements in the Code of Federal Regulations and with the FDA for definitive guidance.

^b^As long as the device is not labeled as sterile, it is exempt from CGMP QS requirements except for 21 C.F.R. § 820.180 (records) and 21 C.F.R. § 820.198 (complaint files).

^c^See Food and Drug Administration. *Guidance for Industry and Food and Drug Administration Staff. Distribution of* In Vitro *Diagnostic Products Labeled for Research Use Only or Investigational Use Only*. US Department of Health and Human Services. November 25, 2013.^38^

^d^Medical device reporting 21 C.F.R. § 803 is specifically mentioned in Food and Drug Administration. *Commercially Distributed Analyte Specific Reagents (ASRs): Frequently Asked Questions. Guidance for Industry and FDA Staff.* September 2007.^39^

^e^See 21 C.F.R. § 864.4020 for blood banking special controls.

^f^There are requirements unique to ASRs used in assays for “contagious condition[s]” that are “highly likely to result in a fatal outcome” and in “donor screening for conditions . . . to safeguard the blood supply” (see 21 C.F.R. § 864.4020).

^g^See Food and Drug Administration. *FDA Guidance for Submission of Immunohistochemistry Applications to the FDA*. 1998.^34^

^h^Only Class I IHC devices are listed as exempt in FDA table at https://www.accessdata.fda.gov/scripts/cdrh/cfdocs/cfpcd/315.cfm?GMPPart=864 (accessed January 3, 2025).

^i^Limitations described in 21 C.F.R. § 864.9.

^j^Exempt from CGMP QS requirements except for 21 C.F.R. § 820.180 (records) and 21 C.F.R. § 820.198 (complaint files).

^k^21 C.F.R. § 864.5200 describes as Class II (performance standards) but does not list specific special controls.

^l^See Class II special controls guidance document Food and Drug Administration. *Premarket Notifications for Automated Differential Cell Counters for Immature or Abnormal Blood Cells; Final Guidance for Industry and FDA*. December 4, 2001.

^m^Special controls listed at 21 C.F.R. § 864.7010.

^n^Special controls listed at 21 C.F.R. § 864.3700.

^o^Special controls listed at 21 C.F.R. § 864.3750.

Most of the product codes associated with anatomic pathology are found under the Hematology and Pathology Devices medical specialty category (21 C.F.R. § 864), whereas a smaller number can be found under the Immunology and Microbiology Devices medical specialty category (21 C.F.R. § 866), including many molecular diagnostic assays. Selection of an appropriate product code is critically important in medical device listing and the premarket review process, as device categories dictate applicable Class I, II, or III requirements for which the device will be assessed. This will be a significant endeavor associated with LDT Final Rule stage 2, 4, and 5 requirements.

### Digital Pathology and Artificial Intelligence

There are now unique product codes and a technical guidance document associated with digital pathology. In 2016, the FDA issued a guidance document, Technical Performance Assessment of Digital Pathology Whole Slide Imaging Devices, intended for manufacturers to use in assessing the technical characteristics of such devices.^55^ A year later and through a De Novo premarket review, the first whole-slide imaging system was approved by the FDA for the “review and interpretation” of digital slides from biopsied tissue.^56^ Special controls specific for whole-slide imaging systems as well as for software algorithm devices to assist users in digital pathology (which are classified as Class II medical devices) are also incorporated into the Code of Federal Regulations (see 21 C.F.R. § 864.3700). The number of whole-slide imaging devices receiving clearance from the FDA has been increasing recently, with nine substantial equivalence determinations from the FDA in 2024 alone.^57^ Significant questions remain in the clinical laboratory community, however, regarding the regulatory requirements associated with changing components of FDA-cleared systems (eg, monitors), and these issues are particularly pertinent where digital pathology is being implemented in hybrid and remote practice settings. The number of artificial intelligence (AI)–based software programs designed to assist anatomic pathologists will also continue to expand since the first FDA approval in 2021.^58^ Product codes particularly relevant to digital pathology include “PSY” (whole-slide imaging systems), “PZZ” (digital pathology display), “QKQ” (digital pathology image viewing and management software), “QPN” (software algorithm device to assist users in digital pathology), and “QYV” (digital cervical cytology slide imaging system with AI) (see also **Supplementary Appendix S1**).

### FDA User Fees

FDA submissions are subject to user fees negotiated as part of MDUFA and are reauthorized every five years. For example, the fiscal year 2025 user fees include annual establishment registration fees of $9,280, as well as 510(k) submission fees of $24,335, PMA submission fees of $540,783, and De Novo classification request fees of $162,235.^59^ There are discounts available for small businesses with gross receipts or sales of less than or equal to $100 million annually.^59^ These submission fees are per assay, and both the annual establishment registration fee and the submission fees for new LDTs are applicable to clinical laboratories under the LDT Final Rule starting in stage 2 (registration) and stages 4/5 (premarket review). Significant additional expenses contribute to the total cost of developing and submitting an IVD to the FDA. The FDA’s LDT Final Rule Regulatory Impact Analysis provides primary estimates for the anticipated cost of compliance by developers (per test) at $4.4 million for a PMA and $0.25 million for a 510(k) with method comparison studies.^60^ External survey data suggest the FDA’s primary estimates are far lower than what manufacturers are actually spending on these activities.^61^ Regardless, these costs far exceed potential resources available to nearly all clinical laboratory settings.

## NYS CLEP AND LDTs

NYS CLEP has received significant attention due to the FDA’s consideration of NYS CLEP–approved LDTs under its category of partial enforcement discretion. As background, a New York permit and compliance with New York clinical laboratory standards are required of all clinical laboratories that perform testing on New York patients. Furthermore, NYS CLEP also has an LDT test approval process that permitted laboratories must comply with before offering clinical testing with an LDT. Conversely, tests that are FDA cleared/approved are not required to follow the NYS CLEP LDT test approval process.

Relevant to anatomic pathology, NYS CLEP has an additional category of clinical laboratory testing called “standard method,” which is defined as a “protocol that is universally applied in laboratories that employ the method for the analyte, such application being consistent with industry standards recognized by leading authorities in laboratory science . . . in general, methods in use prior to FDA’s 1976 Medical Device Amendments.”^62^ Examples of standard methods provided by NYS CLEP on their website include “immunohistochemical (IHC) stains and classic in-situ hybridization (ISH) probes used for tissue pathology . . . when used as part of MD pathologist-guided processes under cytopathology and histopathology.”^62^ Of note, NYS CLEP does not require such standard methods to be submitted as part of the LDT test approval process, although testing with standard methods still requires a New York laboratory permit and the applicable permit category. To date, NYS CLEP has not provided clarification on whether the addition of automation impacts their assessment of whether methods such as IHC or ISH are still considered standard methods.

## DISCUSSION

The LDT Final Rule establishes significant additional requirements and regulatory costs applicable to most clinical laboratories performing LDTs, including those focused on and/or performing testing associated with anatomic pathology. While the topic of anatomic pathology was only addressed in a small number of public comments associated with the LDT Final Rule, this should not be misconstrued as the LDT Final Rule not applying to anatomic pathology. The FDA’s 1976-type LDT targeted enforcement discretion policy does lessen the regulatory burden for laboratories still performing manual IHC and ISH. However, as IHC and ISH are now automated by most clinical laboratories, this targeted enforcement discretion policy will not be applicable in many settings. Moreover, the high number of currently performed types of IHC and ISH tests (from tens at smaller pathology practices to hundreds at academic centers and reference laboratories), as well as the ongoing expansion of the test menus in response to increasing diagnostic and therapeutic complexity, poses an ongoing significant financial burden on clinical laboratories. Similarly, the FDA’s unmet needs partial enforcement discretion policy may reduce regulatory burden in certain contexts (eg, in testing for patients within that healthcare system when FDA-cleared/approved alternatives do not exist), but that provision does not apply for many other important use cases, including outreach testing and LDTs performed by reference laboratories. This severely limits the applicability of the unmet needs provision, particularly in testing for rare disorders, which has the potential to exacerbate an already underserved area within medicine for patients who may have extended journeys toward a correct diagnosis.

To prepare for compliance with the LDT Final Rule, pathologists and laboratory directors, managers, and supervisors (including those overseeing anatomic pathology testing) should understand the FDA’s 5-stage phase-out policy and recognize the associated deadlines (see **FIGURE 1**). Certain requirements (eg, MDR and complaint files requirements outlined in stage 1) necessitate development of written procedures before the implementation date (May 6, 2025). Readers should consult with educational webinars and resources focused on the Final Rule and stage 1 compliance for details on meeting this first set of requirements. It should also be emphasized that in the context of pending litigation against the LDT Final Rule, uncertainty regarding litigation outcome and timing, and the change in regulatory and legislative priorities of a new administration and Congress, making strategic and resource-constrained decisions regarding the LDT Final Rule will be challenging. However, preparation is essential for ensuring compliance with applicable federal regulations, and at the time of this article’s preparation, the LDT Final Rule still carries the full force and effect of law in the United States.

The complexity of QS/CGMP requirements applicable to IVDs **TABLE 1**, as well as the subset of these requirements that are applicable to LDTs developed and performed in CLIA high-complexity settings in accordance with stage 3 of the Final Rule (see **FIGURE 1**), will be new to most clinical laboratory settings. Understanding both the meaning and obligation of these requirements and how they should be applied going forward under the LDT Final Rule will be essential to making effective decisions regarding current and potential future LDT development. Similarly, familiarity with medical device classification and product codes will also be essential for clinical laboratories’ ability to adhere to stage 2 (ie, listing and labeling) and stage 4 and 5 (ie, premarket review) requirements under the LDT Final Rule. Readers should explore the device classification resources referenced in this article and, more specifically, in the context of anatomic pathology, as well as review the classifications and supplemental product codes outlined in **Supplementary Appendix S1** to gain a familiarity with how your LDTs may fit within existing classifications. Application of appropriate product codes will be critical to ensuring that the appropriate controls are applied to new LDTs going forward. The matrix of applicable regulations by product classification can be very complex (see **TABLE 5**), but familiarity with and review of applicable regulations can help clarify specific requirements. The FDA presubmission (Pre-Sub) and Q-Submission (Q-Sub) formal question submission process can also be used to solicit feedback from the FDA on specific questions.^63^

At the time of this article’s preparation, there are two active lawsuits contesting the LDT Final Rule and the FDA’s asserted statutory authority to regulate LDTs—one from the American Clinical Laboratory Association^64^ and the other from the Association for Molecular Pathology.^65^ Additionally, with the 2024 presidential and congressional elections now behind us, the executive branch and both chambers of Congress during this term will be controlled by one party that traditionally has been averse to expanding authority of federal agencies. The new administration may certainly have different priorities for the Department of Health and Human Services, and it is within the authority of the new administration to establish new rules through notice and comment rulemaking to advance its regulatory priorities and/or eliminate previously established rules that it does not support. The timing and outcome of litigation around the LDT Final Rule is still uncertain, and it should be emphasized that as long as the LDT Final Rule continues to have the full force and effect of law in the United States, understanding its requirements is essential for all US clinical laboratories that are preparing to comply with these requirements. Professional organizations within pathology and laboratory medicine will continue to update the community on the status of litigation, rulemaking, and future legislative proposals.

Regardless of the outcome of litigation in opposition to the LDT Final Rule and the new administration’s stance on potential FDA authority over LDTs, it is likely that future congressional IVD and LDT reform efforts will occur. It is possible (if not probable) that similar or related bills to the VALID Act will be introduced in a future congressional session and could encode in statutory text similar requirements and regulatory burdens as does the LDT Final Rule. From an advocacy perspective, it is imperative for members of the clinical laboratory community to understand the proposed content and implications of any legislative LDT and/or IVD oversight reform proposals. Providing substantial feedback during the process is essential, as laws passed by Congress bestow definitive requirements that must be followed to avoid civil and/or criminal penalties. As such, legislative oversight proposals should reflect a regulatory framework that clinical laboratories can practically, logistically, and financially comply with. Our ability to support patient care and diagnostic innovation depends on the outcome of, and meaningful participation in, these efforts.

## Supplementary Material

aqae181_suppl_Supplementary_Appendixs
