# Supplementary material for: Laboratory-developed tests and in vitro diagnostics: A regulatory overview for anatomic pathology
Source: Am J Clin Pathol. 2025 Feb 6;163(5):730–43. doi: 10.1093/ajcp/aqae181 (PMC12086060; doi:10.1093/ajcp/aqae181)
Supplement: aqae181_suppl_Supplementary_Appendixs [file aqae181_suppl_supplementary_appendixs.pdf]

Supplemental Appendix S1. Genzen et al. *Laboratory-Developed Tests and In Vitro Diagnostics - A Regulatory Overview for Anatomic Pathology.*

For up-to-date product classifications, please consult the FDA product code classification database at: https://www.fda.gov/medical-devices/classify-your-medical-device/product-code-classification-database. Product codes lists below were downloaded and organized in October and November 2024 from the FDA product code classification database.

PART 864 -- HEMATOLOGY AND PATHOLOGY DEVICES
Subpart B - Biological Stains
Sec. 864.1860 Immunohistochemistry reagents and kits.

PRODUCT CODE	DEVICE NAME	CLASS	THIRD PARTY ELIGIBLE	REGULATION NUMBER
MXZ	Immunohistochemistry Assay, Antibody, Progesterone Receptor	2	Y	864.1860
MYA	Immunohistochemistry Antibody Assay, Estrogen Receptor	2	Y	864.1860
NBK	System, Test, (Ihc), Tumor Marker, Monitoring, Bladder Cancer	2	N	864.1860
NJT	Immunohistochemistry Reagents And Kits	1	N	864.1860
NJW	Control Material, Her-2/Neu, Immunohistochemistry	2	Y	864.1860
NKF	Immunohistochemistry Antibody Assay, C-Kit	3	N	864.1860
NOT	Microscope, Automated, Image Analysis, Operator Intervention	2	N	864.1860
NQF	Immunohistochemistry Assay, Antibody, Epidermal Growth Factor Receptor	3	N	864.1860
NQN	Microscope, Automated, Image Analysis, Immunohistochemistry,Operator Intervention,Nuclear Intensity & Percent Positivity	2	N	864.1860
NTR	Immunohistochemical Reagent, Antibody (Monoclonal Or Polyclonal) To P63 Protein In Nucleus Of Prostatic Basal Cells	1	N	864.1860
OEO	Automated Digital Image Manual Interpretation Microscope	2	N	864.1860
QNH	Immunohistochemistry Test, Dna Mismatch Repair (Mmr) Protein Assay	3	N	864.1860
QUL	Immunohistochemistry Assay, Antibody, Folr1	3	N	864.1860
QZJ	Immunohistochemistry assay, antibody, claudin 18	3	N	864.1860

Note: Product codes LPI and LPJ omitted from the table due to chemistry review panel classification status.

OTHER ANTIBODIES

PRODUCT CODE	DEVICE NAME	CLASS	THIRD PARTY ELIGIBLE	REGULATION NUMBER
OWF	Immunohistochemical Assay, Helicobacter Pylori	1	Y	866.3110
PKW	Immunohistochemistry Assay, Antibody, Anaplastic Lymphoma Kinase	3	N	
PLS	Immunohistochemistry Assay, Antibody, Programmed Death-Ligand 1	3	N	
QQT	Immunohistochemistry Assay, Antibody, Ki-67	3	N	

PART 864 -- HEMATOLOGY AND PATHOLOGY DEVICES
Subpart B - Biological Stains
Sec. 864.1850 Dye and chemical solution stains.

PRODUCT CODE	DEVICE NAME	CLASS	THIRD PARTY ELIGIBLE	REGULATION NUMBER
GJH	Stain, Reticulocyte	1	N	864.18500
GJJ	Stains, Heinz Body	1	N	864.18500
GJL	Romanowsky Stains	1	N	864.18500
JTS	Stains, Microbiologic	1	N	864.18500
GGH	Iron Stains	1	N	864.18500
GHZ	Solution, Gugol Blue	1	N	864.18500
HYB	Eosin Y	1	N	864.18500
HYC	Fast Green	1	N	864.18500
HYD	Fast Red Salt B	1	N	864.18500
HYE	Solution, Fontanna Silver	1	N	864.18500
HYF	Giemsa Stain	1	N	864.18500
HYG	Glenner'S Stain	1	N	864.18500
HYH	Gold Chloride	1	N	864.18500
HYI	Iodine, Grams	1	N	864.18500
HYJ	Hematoxylin	1	N	864.18500
HYK	Hematoxylin Harris's	1	N	864.18500

HYL	Hematoxylin Mayer's	1	N	864.18500
HYO	Hematoxylin Weigert's	1	N	864.18500
HYQ	Iron Chloride-Weigert	1	N	864.18500
HYR	Leuco-Patent Blue	1	N	864.18500
HYS	Light Green	1	N	864.18500
HYT	Luxol Fast Blue	1	N	864.18500
HYW	Stain, Trichrome, Mallory's	1	N	864.18500
HYY	Metanil Yellow	1	N	864.18500
HYZ	Methenamine Silver	1	N	864.18500
HZA	Methyl Green	1	N	864.18500
HZC	Mucicarmine	1	N	864.18500
HZD	Muller'S Colloidal Iron	1	N	864.18500
HZE	Nile Blue	1	N	864.18500
HZF	Nuclear Fast Red	1	N	864.18500
HZG	Oil Red O	1	N	864.18500
HZH	Orange G	1	N	864.18500
HZJ	Stain, Papanicolau	1	N	864.18500
HZL	Phloxine B	1	N	864.18500
HZM	Hematoxylin, Acid, Phosphotungstic	1	N	864.18500
HZN	Picro Methyl Blue	1	N	864.18500
HZO	Stain, Ponceau	1	N	864.18500
HZP	Pyronin	1	N	864.18500
HZQ	Red-Violet Lb	1	N	864.18500
HZR	Resorcin Fuchsin	1	N	864.18500
HZS	Safranin	1	N	864.18500
HZT	Reagent, Schiff	1	N	864.18500
HZX	Silver Nitrate	1	N	864.18500
HZY	Sirius Red	1	N	864.18500
HZZ	Sudan Black B	1	N	864.18500
IAA	Titan Yellow	1	N	864.18500
IAB	Toluidine Blue	1	N	864.18500
IAC	Van Gieson's Stain	1	N	864.18500
IAD	Van Gieson's Picro-Fuchsin	1	N	864.18500
IAE	Hematoxylin, Iron, Weigert's	1	N	864.18500
IAF	Wright'S Stain	1	N	864.18500
ICC	Eosin B	1	N	864.18500
ICD	Darrow Red	1	N	864.18500
ICF	Crystal Violet For Histology	1	N	864.18500
ICG	Cresyl Violet Acetate	1	N	864.18500
ICH	Congo Red	1	N	864.18500
ICI	Hematoxylin, Chrome Alum	1	N	864.18500
ICJ	Carbol Night Blue	1	N	864.18500
ICL	Carbol Fuchsin	1	N	864.18500
ICM	Brilliant Yellow	1	N	864.18500
ICN	Biebrich Scarlet	1	N	864.18500
ICO	Best's Carmine	1	N	864.18500
ICQ	Azure A	1	N	864.18500
ICR	Azocarmine B	1	N	864.18500
ICS	Azocarmine G	1	N	864.18500
ICT	Azan Counterstain	1	N	864.18500
ICX	Aniline	1	N	864.18500
ICY	Acid, Aniline, Fuchsin	1	N	864.18500
ICZ	Ammoniacal Silver Hydroxide Silver Nitrate	1	N	864.18500
IDA	Alcian Blue	1	N	864.18500
IDB	Aldehyde Fuchsin	1	N	864.18500
IDC	Acridine Orange	1	N	864.18500
IDD	Alizarin Red	1	N	864.18500
IDE	Acid, Hematein	1	N	864.18500
IDF	Acid, Fuchsin	1	N	864.18500

JCH	Esterase	1	N	864.18500
JCI	Acid Phosphatase, Cytochemical	1	N	864.18500
KFC	Methylene Blue, Tissue Stain	1	N	864.18500
KFD	Aniline Blue	1	N	864.18500
KFE	Neutral Red	1	N	864.18500
KJK	Auramine O	1	N	864.18500
KJL	Azure C	1	N	864.18500
KJM	Bismarck Brown Y	1	N	864.18500
KJN	Brilliant Cresyl Blue	1	N	864.18500
KJO	Brilliant Green	1	N	864.18500
KJP	Carmine	1	N	864.18500
KJQ	Chlorazol Black E	1	N	864.18500
KJR	Erythrosin B	1	N	864.18500
KJS	Ethyl Eosin	1	N	864.18500
KJT	Indigocarmine	1	N	864.18500
KJW	Janus Green B	1	N	864.18500
KJX	Jenner Stain	1	N	864.18500
KJY	Malachite Green	1	N	864.18500
KJZ	Martius Yellow	1	N	864.18500
KKA	Methyl Orange	1	N	864.18500
KKB	Methyl Violet 2b	1	N	864.18500
KKC	Methylene Violet	1	N	864.18500
KKD	Nigrosin	1	N	864.18500
KKE	Orange Ii	1	N	864.18500
KKF	Orcein	1	N	864.18500
KKG	Protargol S	1	N	864.18500
KKH	Tablet, Resazurin	1	N	864.18500
KKI	Rose Bengal	1	N	864.18500
KKJ	Sudan Iii	1	N	864.18500
KKK	Sudan Iv	1	N	864.18500
KKL	Thionin	1	N	864.18500
KKM	Methylene Blue Thiocyanate	1	N	864.18500
KKP	Solution, Silver Carbonate	1	N	864.18500
KKQ	Sodium Periodate	1	N	864.18500
KKR	Potassium Periodate	1	N	864.18500
KKS	Acid, Periodic	1	N	864.18500
KKT	Hematoxylin, Ehrlich's	1	N	864.18500
KKW	Basic Fuchsin	1	N	864.18500
KQC	Stains, Hematology	1	N	864.18500
LED	Stains, Chemical Solution	1	N	864.18500
LEE	Stains, Dye Solution	1	N	864.18500
LEF	Stains, Dye Powder	1	N	864.18500
LGY	Trypan Blue	1	N	864.18500

PART 864 -- HEMATOLOGY AND PATHOLOGY DEVICES
Subpart B - Biological Stains
Sec. 864.1865 Cervical intraepithelial neoplasia (CIN) test system.

PRODUCT CODE	DEVICE NAME	CLASS	THIRD PARTY ELIGIBLE	REGULATION NUMBER
PRB	Cervical Intraepithelial Neoplasia (Cin) Test System	2	N	864.1865

PART 864 -- HEMATOLOGY AND PATHOLOGY DEVICES
Subpart B - Biological Stains
Sec. 864.1866 Lynch syndrome test systems.

PRODUCT CODE	DEVICE NAME	CLASS	THIRD PARTY ELIGIBLE	REGULATION NUMBER
PZJ	Lynch Syndrome Test System	2	N	864.1866

PART 864 -- HEMATOLOGY AND PATHOLOGY DEVICES
Subpart B - Biological Stains
Sec. 864.1870 Early growth response 1 (EGR1) gene fluorescence in-situ hybridization (FISH) test system for specimen characterization.

PRODUCT CODE	DEVICE NAME	CLASS	THIRD PARTY ELIGIBLE	REGULATION NUMBER
PDO	Early Growth Response Gene 1 (Egr1) Fish Probe Kit For Specimen Characterization	2	Y	864.1870
PFG	Dna Fish Probe Kit For Specimen Characterization, Human Chromosome, Hematological Disorders	2	Y	864.1870

PART 864 -- HEMATOLOGY AND PATHOLOGY DEVICES
Sec. 864.1880 Fish based detection of chromosomal abnormalities from patients with hematologic malignancies

PRODUCT CODE	DEVICE NAME	CLASS	THIRD PARTY ELIGIBLE	REGULATION NUMBER
QDI	Fish Based Detection Of Chromosomal Abnormalities From Patients With Hematologic Malignancies	2	Y	864.1880

PART 864 -- HEMATOLOGY AND PATHOLOGY DEVICES
Subpart C - Cell And Tissue Culture Products
Sec. 864.2220 Synthetic cell and tissue culture media and components.

PRODUCT CODE	DEVICE NAME	CLASS	THIRD PARTY ELIGIBLE	REGULATION NUMBER
KIT	Media And Components, Synthetic Cell And Tissue Culture	1	N	864.2220

PART 864 -- HEMATOLOGY AND PATHOLOGY DEVICES
Subpart C - Cell And Tissue Culture Products
Sec. 864.2240 Cell and tissue culture supplies and equipment.

PRODUCT CODE	DEVICE NAME	CLASS	THIRD PARTY ELIGIBLE	REGULATION NUMBER
NVG	Tissue Culture, Accessories, Dental	1	N	864.2240
KIY	Chamber, Slide Culture	1	N	864.2240
KIZ	Dish, Tissue Culture	1	N	864.2240
KJA	Flask, Tissue Culture	1	N	864.2240
KJB	Apparatus, Roller	1	N	864.2240
KJC	Bottle, Roller, Tissue Culture	1	N	864.2240
KJD	Spinner, Flask	1	N	864.2240
KJE	Spinner System, Cell Culture	1	N	864.2240
KJF	System, Suspension, Cell Culture	1	N	864.2240
KJH	Apparatus, Perfusion	1	N	864.2240

PART 864 -- HEMATOLOGY AND PATHOLOGY DEVICES
Subpart C - Cell And Tissue Culture Products
Sec. 864.2260 Chromosome culture kit.

PRODUCT CODE	DEVICE NAME	CLASS	THIRD PARTY ELIGIBLE	REGULATION NUMBER
KIQ	Kit, Culture, Chromosome	1	N	864.2260

PART 864 -- HEMATOLOGY AND PATHOLOGY DEVICES
Subpart C - Cell And Tissue Culture Products
Sec. 864.2280 Cultured animal and human cells.

PRODUCT CODE	DEVICE NAME	CLASS	THIRD PARTY ELIGIBLE	REGULATION NUMBER
KIR	Cells, Animal And Human, Cultured	1	N	864.2280

PART 864 -- HEMATOLOGY AND PATHOLOGY DEVICES
Subpart C - Cell And Tissue Culture Products
Sec. 864.2360 Mycoplasma detection media and components.

PRODUCT CODE	DEVICE NAME	CLASS	THIRD PARTY ELIGIBLE	REGULATION NUMBER
KIW	Kit, Mycoplasma Detection	1	N	864.2360
KIX	Media, Mycoplasma Detection	1	N	864.2360
KPB	Media And Components, Mycoplasma Detection	1	N	864.2360

PART 864 -- HEMATOLOGY AND PATHOLOGY DEVICES
Subpart C - Cell And Tissue Culture Products
Sec. 864.2800 Animal and human sera.

PRODUCT CODE	DEVICE NAME	CLASS	THIRD PARTY ELIGIBLE	REGULATION NUMBER
KIS	Sera, Animal And Human	1	N	864.2800

PART 864 -- HEMATOLOGY AND PATHOLOGY DEVICES
Subpart C - Cell And Tissue Culture Products
Sec. 864.2875 Balanced salt solutions or formulations.

PRODUCT CODE	DEVICE NAME	CLASS	THIRD PARTY ELIGIBLE	REGULATION NUMBER
KIP	Formulations, Balanced Salt Solutions	1	N	864.2875

PART 864 -- HEMATOLOGY AND PATHOLOGY DEVICES
Subpart D - Pathology Instrumentation and Accessories
Sec. 864.3010 Tissue processing equipment.

PRODUCT CODE	DEVICE NAME	CLASS	THIRD PARTY ELIGIBLE	REGULATION NUMBER
MXT	Device, Accessory, Cooling Plate For Tissue Embedding Station	1	N	864.3010
IDL	Microtome, Accessories	1	N	864.3010
IDM	Microtome, Ultra	1	N	864.3010
IDN	Microtome, Freezing Attachment	1	N	864.3010
IDO	Microtome, Rotary	1	N	864.3010
IDP	Microtome, Cryostat	1	N	864.3010
IDQ	Infiltrator	1	N	864.3010
IDR	Ovens, Paraffin	1	N	864.3010
IDS	Melting Pot, Paraffin	1	N	864.3010
IDT	Apparatus, Melting Point, Paraffin	1	N	864.3010
IDW	Dispensers, Paraffin	1	N	864.3010
IDX	Sieves, Tissue	1	N	864.3010
IDY	Bath, Flotation, Tissue	1	N	864.3010
IDZ	Cassettes, Tissue	1	N	864.3010
IEG	Table, Slide Warming	1	N	864.3010
IEH	Lamps, Slide Warming	1	N	864.3010
IHJ	Blender, Sputum	1	N	864.3010
KDZ	Device, Decalcifier, Electrolytic	1	N	864.3010
KER	Container, Embedding	1	N	864.3010
KES	Coverslips, Microscope Slide	1	N	864.3010
KET	Filters, Cell Collection, Tissue Processing	1	N	864.3010
KEW	Slides, Microscope	1	N	864.3010
KFL	Microtome, Sliding	1	N	864.3010
KJG	Tube, Tissue Culture	1	N	864.3010
LEC	Grinder, Tissue	1	N	864.3010
MJI	Specimen/Tissue, Identification Orientation	1	N	864.3010

PART 864 -- HEMATOLOGY AND PATHOLOGY DEVICES
Subpart D - Pathology Instrumentation and Accessories
Sec. 864.3250 Specimen transport and storage container.

PRODUCT CODE	DEVICE NAME	CLASS	THIRD PARTY ELIGIBLE	REGULATION NUMBER
OHZ	Urine Transport Kit (Excludes Hiv Testing)	1	N	864.3250
OIA	Neonatal Blood Collection Kit And Screening Form (Excludes Hiv Testing)	1	N	864.3250
OIB	Blood And Urine Collection Kit (Excludes Hiv Testing)	1	N	864.3250

OIE	Urine Collection Kit (Excludes Hiv Testing)	1	N	864.3250
FMH	Container, Specimen, Sterile	1	N	864.3250
NNI	Container, Specimen, Non-Sterile	1	N	864.3250
KDT	Container, Specimen Mailer And Storage, Sterile	1	N	864.3250
KDW	Container, Specimen Mailer And Storage, Temperature Controlled, Sterile	1	N	864.3250
NNK	Container, Specimen Mailer And Storage, Non-Sterile	1	N	864.3250
NNL	Container, Specimen Mailer And Storage, Temperature Controlled, Non-Sterile	1	N	864.3250

PART 864 -- HEMATOLOGY AND PATHOLOGY DEVICES
Subpart D - Pathology Instrumentation and Accessories
Sec. 864.3260 OTC test sample collection systems for drugs of abuse testing.

PRODUCT CODE	DEVICE NAME	CLASS	THIRD PARTY ELIGIBLE	REGULATION NUMBER
MPQ	Container, Specimen, Urine, Drugs Of Abuse, Over The Counter	1	N	864.3260

PART 864 -- HEMATOLOGY AND PATHOLOGY DEVICES
Subpart D - Pathology Instrumentation and Accessories
Sec. 864.3300 Cytocentrifuge.

PRODUCT CODE	DEVICE NAME	CLASS	THIRD PARTY ELIGIBLE	REGULATION NUMBER
IFB	Cytocentrifuge	1	N	864.3300

PART 864 -- HEMATOLOGY AND PATHOLOGY DEVICES
Subpart D - Pathology Instrumentation and Accessories
Sec. 864.3400 Device for sealing microsections.

PRODUCT CODE	DEVICE NAME	CLASS	THIRD PARTY ELIGIBLE	REGULATION NUMBER
KIM	Device For Sealing Microsections	1	N	864.3400

PART 864 -- HEMATOLOGY AND PATHOLOGY DEVICES
Subpart D - Pathology Instrumentation and Accessories
Sec. 864.3600 Microscopes and accessories.

PRODUCT CODE	DEVICE NAME	CLASS	THIRD PARTY ELIGIBLE	REGULATION NUMBER
IBJ	Light, Microscope	1	N	864.3600
IBK	Microscope, Fluorescence/U.V.	1	N	864.3600
IBL	Microscope, Inverted Stage, Tissue Culture	1	N	864.3600
IBM	Microscope, Phase Contrast	1	N	864.3600
KEG	Lamps, Microscope	1	N	864.3600
KEH	Micrometers, Microscope	1	N	864.3600
KEI	Condensers, Microscope	1	N	864.3600
KEJ	Stages, Microscope	1	N	864.3600

PART 864 -- HEMATOLOGY AND PATHOLOGY DEVICES
Subpart D - Pathology Instrumentation and Accessories
Sec. 864.3700 Whole slide imaging system.

PRODUCT CODE	DEVICE NAME	CLASS	THIRD PARTY ELIGIBLE	REGULATION NUMBER
PSY	Whole Slide Imaging System	2	N	864.3700
PZZ	Digital Pathology Display	2	Y	864.3700
QKQ	Digital Pathology Image Viewing And Management Software	2	N	864.3700

PART 864 -- HEMATOLOGY AND PATHOLOGY DEVICES
Subpart D - Pathology Instrumentation and Accessories
Sec. 864.3750 Software algorithm device to assist users in digital pathology.

PRODUCT CODE	DEVICE NAME	CLASS	THIRD PARTY ELIGIBLE	REGULATION NUMBER
QPN	Software Algorithm Device To Assist Users In Digital Pathology	2	N	864.3750

PART 864 -- HEMATOLOGY AND PATHOLOGY DEVICES
Subpart D - Pathology Instrumentation and Accessories
Sec. 864.3800 Automated slide stainer.

PRODUCT CODE	DEVICE NAME	CLASS	THIRD PARTY ELIGIBLE	REGULATION NUMBER
KEY	Stainer, Tissue, Automated	1	N	864.3800
KIN	Slide Stainer, Contact Type	1	N	864.3800
KIO	Slide Stainer, Immersion Type	1	N	864.3800
KPA	Slide Stainer, Automated	1	N	864.3800

PART 864 -- HEMATOLOGY AND PATHOLOGY DEVICES
Subpart D - Pathology Instrumentation and Accessories
Sec. 864.3875 Automated tissue processor.

PRODUCT CODE	DEVICE NAME	CLASS	THIRD PARTY ELIGIBLE	REGULATION NUMBER
IEO	Processor, Tissue, Automated	1	N	864.3875

Sec. 864.390 Digital Cervical Cytology Slide Imaging System With Artificial Intelligence

PRODUCT CODE	DEVICE NAME	CLASS	THIRD PARTY ELIGIBLE	REGULATION NUMBER
QYV	Digital Cervical Cytology Slide Imaging System With Artificial Intelligence Algorithm	2	N	864.3900

PART 864 -- HEMATOLOGY AND PATHOLOGY DEVICES
Subpart E - Specimen Preparation Reagents
Sec. 864.4010 General purpose reagent.

PRODUCT CODE	DEVICE NAME	CLASS	THIRD PARTY ELIGIBLE	REGULATION NUMBER
IFO	Solution, Newcomer's	1	N	864.4010
IFS	Solution, Helly	1	N	864.4010
IGK	Solution, Clarke's	1	N	864.4010
IJZ	Oil, Clearing	1	N	864.4010
IBB	Phytohemaglutinin M	1	N	864.4010
KEF	Paraformaldehyde	1	N	864.4010
HZI	Acid, Osmic	1	N	864.4010
IAL	Iodine (Tincture)	1	N	864.4010
IAM	Solution, Lugol's	1	N	864.4010
IAT	Syrup, Gum, Apathy's	1	N	864.4010
IAW	Collodion	1	N	864.4010
IAY	Colchicine	1	N	864.4010
IER	Polyethylene Glycol (Carbowax)	1	N	864.4010
IEX	Gelatin	1	N	864.4010
IEZ	Celloidin	1	N	864.4010
IFF	Solution, Decalcifier, Electrolytic	1	N	864.4010
IFH	Solution, Zenker's	1	N	864.4010
IFI	Sprays, Synthetic, Smear	1	N	864.4010
IFJ	Fixative, Richardson Glycol	1	N	864.4010
IFL	Polyethylene Glycol Preservative	1	N	864.4010
IFN	Solution, Orth's	1	N	864.4010
IFP	Formalin, Neutral Buffered	1	N	864.4010
IFQ	Formulations, Mercuric Chloride For Tissue	1	N	864.4010
IFT	Glutaraldehyde	1	N	864.4010
IFY	Gelatin-Formalin	1	N	864.4010
IFZ	Gelatin For Specimen Adhesion	1	N	864.4010
IGB	Solution, Formalin-Sodium Acetate	1	N	864.4010
IGC	Formalin-Saline	1	N	864.4010
IGD	Solution, Formol Calcium	1	N	864.4010
IGE	Solution, Formalin Ammonium Bromide	1	N	864.4010
IGF	Solution, Formalin-Alcohol-Acetic Acid	1	N	864.4010

IGG	Formaldehyde (Formalin, Formol)	1	N	864.4010
IGM	Solution, Carnoy's	1	N	864.4010
IGN	Fluid, Bouin's	1	N	864.4010
JCB	Detergent	1	N	864.4010
JCC	Ph Buffer	1	N	864.4010
JCE	Solution, Isotonic	1	N	864.4010
KDX	Solution, Decalcifier, Acid Containing	1	N	864.4010
KDY	Agent, Chelating For Decalcification	1	N	864.4010
KEE	Osmium Tetroxide	1	N	864.4010
KEL	Adhesive, Albumin-Based	1	N	864.4010
KEM	Agent, Clearing	1	N	864.4010
KEO	Formulations, Paraffin, All	1	N	864.4010
KEP	Media, Mounting, Oil Soluble	1	N	864.4010
KEQ	Media, Mounting, Water Soluble	1	N	864.4010
LDW	Fixative, Acid Containing	1	N	864.4010
LDX	Fixative, Metallic Containing	1	N	864.4010
LDY	Fixative, Formalin-Containing	1	N	864.4010
LDZ	Fixative, Alcohol Containing	1	N	864.4010
LEA	Preservative, Cytological	1	N	864.4010
LEB	Mounting Media	1	N	864.4010
PPM	General Purpose Reagent	1	N	864.4010

PART 864 -- HEMATOLOGY AND PATHOLOGY DEVICES
Subpart E - Specimen Preparation Reagents
Sec. 864.4020 Analyte specific reagents.

PRODUCT CODE	DEVICE NAME	CLASS	THIRD PARTY ELIGIBLE	REGULATION NUMBER
MVU	Reagents, Specific, Analyte	1	N	864.4020

PART 864 -- HEMATOLOGY AND PATHOLOGY DEVICES
Subpart E - Specimen Preparation Reagents
Sec. 864.4400 Enzyme preparations.

PRODUCT CODE	DEVICE NAME	CLASS	THIRD PARTY ELIGIBLE	REGULATION NUMBER
IAZ	Heparin	1	N	864.4400
IBA	Streptolysin O	1	N	864.4400
IBC	Diastase	1	N	864.4400
IBD	Hyaluronidase	1	N	864.4400
IBE	Neuramininase (Sialidase)	1	N	864.4400
IBF	Papain	1	N	864.4400
IBG	Trypsin	1	N	864.4400
LIJ	Peroxidase Anti-Peroxidase Immunohistochemical Stain	1	N	864.4400

PART 864 -- HEMATOLOGY AND PATHOLOGY DEVICES
Subpart F - Automated and Semi-Automated Hematology Devices
Sec. 864.5200 Automated cell counter.

PRODUCT CODE	DEVICE NAME	CLASS	THIRD PARTY ELIGIBLE	REGULATION NUMBER
GKL	Counter, Cell, Automated (Particle Counter)	2	Y	864.5200
GKX	Instrument, Automated Platelet Counting	2	Y	864.5200
LKM	Counter, Urine Particle	2	Y	864.5200

PART 864 -- HEMATOLOGY AND PATHOLOGY DEVICES
Subpart F - Automated and Semi-Automated Hematology Devices
Sec. 864.5220 Automated differential cell counter.

PRODUCT CODE	DEVICE NAME	CLASS	THIRD PARTY ELIGIBLE	REGULATION NUMBER
GKZ	Counter, Differential Cell	2	N	864.5220
MAM	Dna-Probe, B And T Lymphocyte	2	Y	864.5220

PRODUCT CODE	DEVICE NAME	CLASS	THIRD PARTY ELIGIBLE	REGULATION NUMBER
OYE	Flow Cytometric Reagents And Accessories.	2	Y	864.5220
PMG	Automated Multicolor Fluorescent Imaging Cytometric Analysis System	2	Y	864.5220
POV	Semen Analysis Device	2	Y	864.5220
LIZ	Assay, T Lymphocyte Surface Marker	2	Y	864.5220
LJD	Assay, B Lymphocyte Marker	2	Y	864.5220
NID	Assay, Proliferation, In Vitro, T Lymphocyte	2	Y	864.5220
PDX	Flow Cytometry Calibrator	2	Y	864.5220

PART 864 -- HEMATOLOGY AND PATHOLOGY DEVICES
Subpart F - Automated and Semi-Automated Hematology Devices
Sec. 864.5260 Automated cell-locating device.

PRODUCT CODE	DEVICE NAME	CLASS	THIRD PARTY ELIGIBLE	REGULATION NUMBER
JOY	Device, Automated Cell-Locating	2	Y	864.5260
LNJ	Analyzer, Chromosome, Automated	2	N	864.5260
SAL	Device, Automated Cell Locating, Bone Marrow	2	N	864.5261

PART 864 -- HEMATOLOGY AND PATHOLOGY DEVICES
Sec. 864.5261 Device, Automated Cell Locating, Bone Marrow

PRODUCT CODE	DEVICE NAME	CLASS	THIRD PARTY ELIGIBLE	REGULATION NUMBER
SAL	Device, Automated Cell Locating, Bone Marrow	2	N	864.5261

PART 864 -- HEMATOLOGY AND PATHOLOGY DEVICES
Subpart H - Hematology Kits and Packages
Sec. 864.7010 Flow cytometric test system for hematopoietic neoplasms.

PRODUCT CODE	DEVICE NAME	CLASS	THIRD PARTY ELIGIBLE	REGULATION NUMBER
PWD	Flow Cytometric Test System For Hematopoietic Neoplasms	2	Y	864.7010

OTHER DEVICES

PRODUCT CODE	DEVICE NAME	CLASS	THIRD PARTY ELIGIBLE	REGULATION NUMBER
NQI	System, Immunomagnetic, Circulating Cancer Cell, Enumeration	2	N	866.6020
NYI	Classifier, Prognostic, Recurrence Risk Assessment, Rna Gene Expression, Breast Cancer	2	N	866.6040
OVQ	Chronic Lymphocytic Leukemia Fish Probe Kit	2	Y	866.6040
OWK	Early Growth Response 1 (Egr) Fish Probe Kit	2	Y	866.6040
OYX	Bcr/Abl1 Monitoring Test	2	N	866.6060
PSU	Jak2 Gene Mutation Detection Test	2	N	866.6070
PZM	Next Generation Sequencing Based Tumor Profiling Test	2	Y	866.6080
SBY	High Throughput Sequencing Based Tumor Profiling Test Of Circulating Cell-Free Nucleic Acids	2	N	866.6085
QAZ	Cancer Predisposition Risk Assessment System	2	N	866.6090
QVU	High Throughput Dna Sequencing For Hereditary Cancer Predisposition Assessment Test System	2	N	866.6095
QDC	Dna-Based Test For Minimal Residual Disease For Hematologic Malignancies	2	N	866.6100
QSA	Circulating Tumor Cell (Ctc) Enrichment Device	2	N	866.6110
QYO	Hematopoietic Cell Enrichment Kit	2	N	866.6120
NTH	System, Automated Scanning Microscope And Image Analysis For Fluorescence In Situ Hybridization (Fish) Assays	2	N	866.4700
OYU	Dna-Probe Kit, Human Chromosome	2	N	866.4700
QDI	Fish Based Detection Of Chromosomal Abnormalities From Patients With Hematologic Malignancies	2	Y	864.1880
NTH	System, Automated Scanning Microscope And Image Analysis For Fluorescence In Situ Hybridization (Fish) Assays	2	N	866.4700
OVQ	Chronic Lymphocytic Leukemia Fish Probe Kit	2	Y	866.6040
OWK	Early Growth Response 1 (Egr) Fish Probe Kit	2	Y	866.6040
NYQ	Chromogenic In Situ Hybridization, Nucleic Acid Amplification, Her2/Neu Gene, Breast Cancer	3	N	n/a
PMI	Fluorescence In Situ Hybridization, Platelet-Derived Growth Factor Receptor, Beta Polypeptide (Pdgfrb), Rearrangement	3	N	n/a
PNK	Fluorescence In Situ Hybridization, Chromosome 17p Deletion (Tp53)	3	N	n/a
NSD	Test, Fluorescence In Situ Hybridization (Fish), For Bladder Cancer Detection And Monitoring For Recurrence	3	N	n/a
NXG	Fluorescence In Situ Hybridization, Topoisomerase Ii Alpha, Gene Amplification And Deletion	3	N	n/a

OWE	Fluorescence In Situ Hybridization, Anaplastic Lymphoma Kinase, Gene Rearrangement	3	N	n/a
PIV	Automated Indirect Immunofluorescence Microscope And Software-Assisted System For Clinical Use	2	Y	866.4750
